# Multimodal classification of Alzheimer's disease and mild cognitive impairment using custom MKSCDDL kernel over CNN with transparent decision-making for explainable diagnosis

**DOI:** 10.1038/s41598-024-52185-2

**Published:** 2024-01-20

**Authors:** V. Adarsh, G. R. Gangadharan, Ugo Fiore, Paolo Zanetti

**Affiliations:** 1 National Institute of Technology Tiruchirappalli, Tiruchirappalli, India; 2 University of Salerno, Fisciano, Italy; 3 University Parthenope, Naples, Italy

**Keywords:** Magnetic resonance imaging, Brain imaging

## Abstract

The study presents an innovative diagnostic framework that synergises Convolutional Neural Networks (CNNs) with a Multi-feature Kernel Supervised within-class-similar Discriminative Dictionary Learning (MKSCDDL). This integrative methodology is designed to facilitate the precise classification of individuals into categories of Alzheimer's Disease, Mild Cognitive Impairment (MCI), and Cognitively Normal (CN) statuses while also discerning the nuanced phases within the MCI spectrum. Our approach is distinguished by its robustness and interpretability, offering clinicians an exceptionally transparent tool for diagnosis and therapeutic strategy formulation. We use scandent decision trees to deal with the unpredictability and complexity of neuroimaging data. Considering that different people's brain scans are different, this enables the model to make more detailed individualised assessments and explains how the algorithm illuminates the specific neuroanatomical regions that are indicative of cognitive impairment. This explanation is beneficial for clinicians because it gives them concrete ideas for early intervention and targeted care. The empirical review of our model shows that it makes diagnoses with a level of accuracy that is unmatched, with a classification efficacy of 98.27%. This shows that the model is good at finding important parts of the brain that may be damaged by cognitive diseases.

## Introduction

Alzheimer's disease (AD) is a deeply impactful, chronic neurological condition that stands as the leading cause of dementia worldwide. It transforms the very fabric of our cognitive functions, affecting memory, thought processes, and behaviour in profound ways. The disease causes beta-amyloid plaques and tau protein tangles to build up in the brain, making a complicated web. These harmful deposits do not just sit there; they actively interfere with the important signalling between neurons. Over time, this leads to a terrible chain of events, including the death of neurons, a loss of cognitive ability, and a loss of the ability to do things. The condition is not only a medical issue but also a complicated system that has not been fully understood^1,2^. AD typically begins with mild memory loss and confusion and gradually progresses to severe dementia and loss of basic bodily functions, eventually leading to death. This progressive neural and synaptic deterioration leads to a range of cognitive and functional deficits, including memory impairment, language difficulties, confusion, and behavioural changes. The impacts of Alzheimer's disease are substantial and can have a considerable influence on the overall quality of daily life of an individual, as well as that of their caregivers and family members. Despite ongoing research efforts, there is currently no known cure for AD, making it a significant public health challenge. It is a complicated and multifaceted disease whose pathogenesis is still unknown. According to recent studies, there may be up to 50 million instances of Alzheimer's disease globally^3,4^.

MCI is a neurodegenerative condition that affects a sizeable portion of the population. MCI is marked by memory loss and is thought to be an early sign of AD. MCI is generally considered to be a transitional state that occurs between healthy ageing and the onset of AD^5,6^. However, the precise boundary between MCI and AD is often unclear, and there may also be changes in healthy ageing that are of interest to neuroscientists^7^. Understanding the complex relationship between MCI, healthy ageing, and AD is crucial to developing effective interventions for this debilitating neurological disease. It is worth noting that not all people with MCI will develop AD^8^. While some individuals with MCI never develop dementia, others stay in the MCI state indefinitely. Identifying people with MCI at an early stage, however, is critical for slowing the development of AD, even though there is presently no cure for this disease. Studies have shown that early treatment can delay the start of AD symptoms, and therapies have been made to control cognitive and behavioural symptoms. MCI and AD diagnosis require precise and dependable indicators, and several advances have been achieved in this area^9–11^.

### Related work

Advancements in AI/ML have enabled rapid detection and confirmation of AD^12^. In the rapidly evolving field of AD diagnosis, several machine learning and deep learning methods have come to the fore, each with unique strengths and limitations. Ortiz et al.^13^ make a significant contribution by applying deep belief networks to 3D patches of Gray Matter images segmented according to the Automated Anatomical Labelling atlas. This approach is particularly noteworthy for its high accuracy rates and Area Under the Curve (AUC) values, indicating a robust ability to classify not just AD patients but also those with MCI. One limitation, however, is the substantial computational power required due to the complexity of deep learning models. Nanni et al.^14^ tackle the issue of the 'curse-of-dimensionality,' a common problem when dealing with high-dimensional MRI feature vectors. They propose a hybrid ensemble approach that integrates Support Vector Machines (SVMs) trained on different texture descriptors with SVMs trained on voxel-based markers. By employing feature selection algorithms for dimensionality reduction, their system achieves a high classification performance on AD. Despite this, the approach does not fully explore the potential of more advanced machine learning techniques like Convolutional Neural Networks. Feng et al.^15^ introduced AD-WTEF, a method that leverages Wavelet Transformation Energy Features (WTEF) to capture subtle energy distribution differences in Structural Magnetic Resonance Imaging (sMRI) for AD classification. This method is designed to overcome the limitations of traditional spatial analysis techniques. While effective, the approach is not without its challenges, including information redundancy due to the non-downsampling nature of the wavelet transformation. Leming et al.^16^ contributed by addressing the challenge of confounding factors in clinical MRI data. Utilising a massive dataset of 467,464 clinical brain MRI scans from the Mass General Brigham healthcare system, they identified 18 significant confounding factors and curated a confounder-free training set for AD and MCI. They then applied an ensemble of 3D ResNet-50 models, achieving an impressive AUC score of 0.82. However, the study's confinement to a single healthcare system raises questions about its broader applicability. In essence, Ortiz et al. and Leming et al. achieve high model performance but require broader validation to confirm their general applicability. In contrast, Nanni et al. and Feng et al. tackle specific issues in the feature space but could benefit from the integration of more advanced machine-learning methods.

Deep Learning (DL) methods are gaining recognition for their ability to improve AD diagnosis. One of the best things about DL methods is that they can find hidden characteristics across many layers independently. Su et al.^17^ developed the Firefly Algorithm for Anomaly Detection (FAAD) and FAAD + Entropy-Conditional adversarial Domain AdaptatioN (CDANE) algorithms, specialised for few-shot cross-site anomaly detection in mental disorders based on fMRI Functional Connectivity (FC). The inclusion of visualisation analysis for discriminative FC and brain regions adds a layer of biological authenticity to our methods, potentially aiding in the discovery of imaging biomarkers. However, the algorithms are limited by their reliance on a few labelled samples from the target domain, which may introduce a degree of uncertainty in settings with highly variable feature distributions. Pan et al.^18^ proposed AD and MCI diagnosis; the Disease-image-Specific Deep Learning (DSDL) framework offers a novel solution to the pervasive issue of incomplete multimodal neuroimaging data. Unlike extant methodologies, DSDL integrates neuroimage synthesis and disease diagnosis, ensuring a diagnosis-oriented approach to data imputation. Comprising a Disease-image-Specific Network and a Feature-consistency Generative Adversarial Network, the framework excels at capturing disease-specific traits from whole-brain scans and imputing missing data seamlessly. However, the effectiveness of the framework is intrinsically linked to the quality of the imputed neuroimages, making it susceptible to variations in the inherent data quality and potentially limiting its generalizability across diverse clinical settings. Basaia et al.^19^ leveraged CNNs to offer a robust, automated diagnostic solution. Capitalising on 3D T1-weighted MRI scans, they achieved exceptional accuracy levels, particularly in distinguishing AD from healthy controls (up to 99%). A distinct advantage lies in the use of a simplified CNN architecture that minimises computational complexity while maximising performance. However, the model’s performance in discerning c-MCI from s-MCI is not as robust as its ability to classify AD or MCI from healthy controls. Additionally, the algorithm's predictive capabilities could be further enhanced by incorporating other types of data, such as PET scans, CSF biomarkers, and neuropsychological scores. Lei et al.^20^ introduced a novel framework for predicting clinical scores in AD using longitudinal MRI data. Unlike traditional approaches that rely on single time-point data, this framework employs multiple time points to enhance prediction accuracy. It consists of a sophisticated ensemble of feature selection, deep polynomial networks for feature encoding, and support vector regression for longitudinal score prediction. However, the model currently uses only longitudinal MRI data from ADNI, overlooking the potential insights from other modalities like fMRI, PET, and DTI. Lian et al.^21^ introduce a Hierarchical, Fully Convolutional Network (H-FCN) for AD diagnosis using sMRI. Unlike existing methods that rely on predetermined informative locations, H-FCN autonomously identifies discriminative local patches and regions in the brain. These are then used for multi-scale feature representations, upon which hierarchical classification models are constructed. However, the study's scope is limited to sMRI data and does not incorporate other imaging modalities or clinical variables. It remains to be seen how the method performs when integrated with other diagnostic markers or when applied to diverse clinical populations. Future work could focus on multimodal integration for a more comprehensive diagnostic tool.

In the current landscape of AD research, there has been a marked increase in the application of multimodal data for the usage of early identification and diagnosis. The integration of data from diverse sources, including but not limited to MRI, genetics, and clinical data, has led to a significant improvement in the accuracy and reliability of indicators for AD. Jain et al.^22^ used a pre-trained CNN, VGG-16, for classification tasks on brain sMRI slices. By employing transfer learning, the study ingeniously bypasses a common limitation in deep learning: the need for large datasets. The VGG-16 model proves adept at extracting relevant features for classifying AD, MCI, and CN states, achieving an impressive 95.73% accuracy on the validation set. However, the transfer learning approach, while innovative, risks potential incongruities between general image features and the specialised features relevant to neurodegenerative diseases. Spasov et al.^23^ aimed to distinguish between MCI patients at elevated risk for AD conversion and those less likely to convert. Leveraging a multi-tasking approach, the model concurrently tackles MCI-to-AD conversion and AD vs. healthy control classification, facilitating a more robust feature extraction for AD prognosis. In terms of predictive power, the model achieved an AUC of 0.925 and a tenfold cross-validated accuracy of 86% in classifying MCI patients. Although the model utilised various input metrics, the warp field characteristics added little to predictive value. To improve data exchange between layers, Wang et al.^24^ used an ensemble of 3D Densely Connected Convolutional Networks (3D-DenseNets). The use of dense connections within the network ensures optimised information flow and gradient propagation, making it more trainable while using fewer parameters—a critical advantage when dealing with limited training data. However, the focus is solely on MRI data, ignoring potential synergies with other modalities or clinical variables. Cheng et al.^25^ introduce a novel Multimodal Manifold-Regularized Transfer Learning (M2TL) method aimed at effectively predicting the conversion from MCI to AD. Unlike conventional approaches that focus solely on the target domain, M2TL leverages both auxiliary domains and unlabelled samples to enhance predictive performance. Furthermore, the inclusion of group sparsity regularisation allows the model to auto-select informative samples, adding robustness to the classifier. However, it primarily focuses on imaging data, bypassing other potentially valuable diagnostic information like genetic or lifestyle factors. Suk et al.^26^ present an innovative methodological framework that merges Deep Auto-Encoder (DAE) with Hidden Markov Models (HMM) for diagnosing MCI through resting-state functional Magnetic Resonance Imaging (rs-fMRI). However, the study's limitations include its focus solely on computational modelling without incorporating other types of diagnostic data. Furthermore, the method's generalizability beyond the datasets used for validation remains untested. Li et al.^27^ introduce a novel approach for diagnosing AD and MCI using structural MRI scans. The method employs multiple cluster Dense Convolutional Neural Networks (DenseNets) to learn localised features of brain images. The approach achieved a remarkable accuracy of 89.5% for AD vs. Normal Control (NC) and 73.8% for MCI vs. NC, outperforming existing methods. However, its limitations include a focus solely on structural MRI, leaving room for future integration of other imaging modalities like PET for a more comprehensive diagnosis. Additionally, the method's applicability to broader datasets beyond the ADNI database used for validation remains unexplored.

The application of explainable artificial intelligence (XAI) methods has made these models more open and easier to understand, which is important for building trust and getting people to use therapies. Essemlali et al.^28^ employed a modified BrainNet CNN on diffusion-weighted MRI (DW-MRI) tractography connectomes to delve into the structural connectomics of AD and MCI. By leveraging the BrainNetCNN for brain image classification paired with XAI techniques, the researchers accentuated brain regions and their interconnections implicated in AD. This work not only reinforces the potential of deep convolution networks in neurodegenerative disease analysis but also establishes a bridge with traditional AD research findings. Using binary categorisation and layer-by-layer data analysis, El-Sappagh et al.^29^ present a highly accurate and interpretable machine-learning model for AD diagnosis and progression detection. Using 11 modalities and data from 1048 subjects, the two-layer model employs a Random Forest (RF) classifier optimised with key biological and clinical markers. The explainability feature addresses a significant gap in clinical uptake, as it makes the model transparent and trustworthy for physicians. Yu et al.^30^ The study presents an innovative framework that integrates attention mechanisms and multi-scale features for enhanced accuracy and explainability in the visual classification of medical images, specifically for AD. However, the model has limitations: it lacks integration of medical domain knowledge, which could refine its predictive capability, and its latent features are weakly supervised due to a scarcity of publicly available pathological annotations, which could potentially overlook crucial pathological locations in the brain. Lombardi et al.^31^ introduce a machine learning (ML) framework with an XAI component that not only classifies subjects into healthy, cognitively impaired, and dementia categories but also provides explainability via SHapley Additive exPlanations (SHAP) values. However, the study focuses on a fixed set of cognitive and clinical indexes, potentially overlooking other important variables. Also, while it addresses variability within diagnostic categories, the model may not fully capture the complexity of different subcategories within the neurodegenerative spectrum. Shojaei et al.^32^ used a 3D Convolutional Neural Network (3D-CNN) model coupled with a genetic algorithm-based Occlusion Map and Backpropagation-based explainability methods employed for AD diagnosis using MRI scans. The model not only achieves a commendable 87% accuracy in fivefold cross-validation but also successfully identifies key brain regions corroborated by existing AD literature, enhancing its reliability and medical relevance. The focus remains on algorithmic accuracy, potentially missing out on the integration of domain-specific medical knowledge.

Our innovative diagnostic framework stands as a significant advancement in the field of AD and MCI detection, offering a compelling combination of precision, robustness, and interpretability that sets it apart from existing methodologies (see Table 1). While many approaches specialise in either diagnostic accuracy or model interpretability, our framework synergises CNNs with the cutting-edge MKSCDDL algorithm to achieve both. Unlike traditional machine learning methods that often require substantial computational resources or lack adaptability across different imaging data types, our model leverages scandent decision trees to accommodate the complexity and variability of neuroimaging data. This feature allows for more individualised assessments, a granularity often missing in other models. Moreover, our approach includes advanced interpretability methods like Local Interpretable Model-agnostic Explanations (LIME) and Class Activation Maps (CAMs), filling the interpretability gap often noted in deep learning methods like 3D CNNs. These interpretability features not only build trust in the diagnostic process but also provide clinicians with actionable insights for early intervention and targeted care. Even within the realm of XAI, where models like MAXNet and BrainNet CNN have made strides in clarifying their decision-making processes, our model goes a step further. It provides a comprehensive diagnostic tool that is both exceptionally accurate—with a classification efficacy of 98.27%—and transparent in its reasoning. This dual strength makes it a particularly valuable asset for clinicians aiming for precise yet understandable diagnostic results. In summary, our model represents a pinnacle of balanced excellence, offering unparalleled diagnostic accuracy without sacrificing the nuances of interpretability and individualised assessment.

**Table 1:** Summary of related works.

	S. No	Authors	Year	Technique	Contribution	Advantages	Limitations
Traditional ML Methods	1	Ortiz et al	2016	Deep Belief Networks	High accuracy and AUC in classifying AD and MCI subjects	High accuracy and robustness in classification	Requires substantial computational resources
	2	Nanni et al	2019	SVM Ensemble	Tackled the 'curse-of-dimensionality' in MRI feature vectors	High classification performance	Limited use of advanced ML techniques like CNNs
	3	Feng et al	2020	AD-WTEF	Captured subtle energy distribution differences in sMRI	Effective in capturing subtle differences	Information redundancy in wavelet transformation
	4	Leming et al	2022	3D ResNet-50 Ensemble	Addressed confounding factors in clinical MRI data	High AUC score and addresses confounding factors	Confined to a single healthcare system
Deep Learning Methods	5	Su et al	2021	FAAD + CDANE	Anomaly detection in fMRI for mental disorders	Superior accuracy and robustness; biological authenticity through visualisation; better than traditional methods	Limited by dependency on few labelled samples; uncertainty in variable feature distributions
	6	Pan et al	2022	DSDL Framework	Multimodal AD and MCI diagnosis	Seamlessly integrates neuroimage synthesis and disease diagnosis; superior performance in both tasks	Effectiveness tied to the quality of imputed neuroimages; this may limit generalizability across diverse clinical settings
	7	Basaia et al	2019	3D T1-weighted CNNs	AD and MCI diagnosis	Exceptional accuracy in distinguishing AD from healthy controls; performs well across multiple MRI protocols	Limited performance in c-MCI vs. s-MCI classification; could benefit from incorporating other data types
	8	Lei et al	2021	Ensemble Learning	Longitudinal AD score prediction	Uses multiple time points for enhanced prediction accuracy; handles data incompleteness	Restricted to ADNI MRI data; does not include other imaging modalities or clinical details
	9	Lian et al	2022	H-FCN	AD diagnosis using sMRI	Autonomously identifies discriminative local patches and regions; shows promising performance in both atrophy localisation and disease diagnosis	Limited to sMRI data; does not incorporate other diagnostic markers or adapt to diverse clinical populations
Multimodal	10	Jain et al	2019	Transfer Learning using VGG-16	AD and its variants diagnosis using sMRI	Bypasses the need for large datasets; high accuracy (95.73%)	Limited to general image features, potentially not capturing disease-specific nuances
	11	Spasov et al	2019	Multi-tasking Deep Learning Model	Developed a model to distinguish between MCI patients at high and low risk for AD conversion	Minimizes data overfitting with fewer parameters; high predictive power	Limited additional value from warp field characteristics
	12	Wang et al	2019	Ensemble of 3D Densely Connected Convolutional Networks	Used dense connections within 3D-DenseNets to improve data exchange between layers	Optimised information flow and gradient propagation; improved trainability	Solely focused on MRI data, ignoring other modalities or clinical variables
	13	Cheng et al	2015	Multimodal Manifold-Regularized Transfer Learning (M2TL)	Proposed M2TL method to predict MCI to AD conversion, achieving 80.1% accuracy on the ADNI database	Incorporates both target and auxiliary domains for improved performance; auto-selects informative samples	Focused only on imaging data, missing other potential diagnostic markers like genetic or lifestyle factors
	14	Suk et al	2016	Deep Auto-Encoder (DAE) with (HMM)	Developed a framework using DAE and HMM for diagnosing MCI through resting-state functional MRI	Unveils complex functional networks and their dynamics; outperforms existing methods	Focuses solely on computational modelling, lacks incorporation of other diagnostic data; generalizability untested
	15	Li et al	2018	Multiple Cluster Dense Convolutional Neural Networks (DenseNets)	Introduced a novel approach for diagnosing AD and MCI using sMRI scans without requiring pre-processing like registration and segmentation	Eliminates the need for rigid pre-processing; high accuracy (89.5% for AD vs. NC and 73.8% for MCI vs. NC.)	Focused solely on structural MRI; lacks integration with other imaging modalities or broader datasets
XAI Methods	16	Essemlali et al	2020	Modified BrainNet CNN on DW-MRI	Investigated structural connectomics of AD and MCI	Identified AD-implicated brain regions; pioneering use of XAI	Limited to CNN's scope; potential biases in tractography data
	17	El-Sappagh et al	2021	Two-layer Random Forest classifier	AD diagnosis and progression detection using 11 modalities	High accuracy; integrates explainability, bridging the gap for clinical applications	Exclusively reliant on available modalities; transparency does not ensure clinical adoption
	18	Yu et al	2022	MAXNet with attention mechanisms	Enhanced visual classification of medical images for AD	Outperforms in accuracy and explainability; suitable for clinical applications	Absence of integrated medical domain knowledge; weakly supervised latent features
	19	Lombardi et al	2022	ML framework with XAI using SHAP	Classifies subjects and provides explainability on AD progression	Offers insights into AD as a continuum; tracks longitudinal changes	Focuses on specific cognitive and clinical indexes; might not capture the full complexity of AD subcategories
	20	Shojaei et al	2023	3D-CNN with Occlusion Map and Backpropagation-based Explainability	AD diagnosis using MRI scans with explainability	High accuracy; identifies key brain regions; addresses the "black box" problem	Requires further validation; potential oversight in integrating domain-specific medical knowledge

The salient contributions in the paper can be summarised as follows:

- The framework uses a CNN and a within-class-similar Discriminative Dictionary Learning method to reduce misclassification by using structural and anatomical similarities between comparable images in the trained set.
- A decision tree mechanism and a transfer learning process are used to check and improve the accuracy of the classification. LIME and CAM are used to make a model that can be understood and whose decisions can be trusted.
- A new classification method is proposed that combines the CNN's end-to-end pixel-wise mapping with the MKSCDDL kernel, using class similarity as matrices to group medical data. This increases the domain-specific information by testing two slices with a set distance on both sides of the comparison.
- To make the model easy to understand, a scandent decision tree is used to check the ground truth with the CNN and fill in missing data in the multimodality dataset.

The remainder of the paper is organised as follows: "Materials and methods" describes the Discriminant Dictionary Learning algorithm and Scandent Decision Tress, the proposed methodology and implementation of the model. "Result analysis" explains the result analysis, followed by the conclusion and future directions in "Conclusions and future directions".

## Materials and methods

### Discriminant dictionary learning

Discriminant dictionary learning (DDL) is a type of machine learning algorithm used for classification tasks. It is a supervised learning method that involves the creation of a dictionary of features that can be used to discriminate between different classes of data. In DDL, the dictionary is learned by minimising a cost function that includes both a reconstruction error term and a discriminant term. The reconstruction error term ensures that the dictionary can accurately represent the input data, while the discriminant term ensures that the dictionary is optimised for the specific classification task at hand. The discriminant term in the cost function is typically based on a measure of the distance between the dictionary atoms of different classes or on the classification error rate of a linear classifier trained on the learned dictionary. By optimising the dictionary for both reconstruction and discriminant performance, DDL can create a set of features that are well-suited for classification tasks.

Consider that $Y={\left\{{{\varvec{y}}}_{{\varvec{i}}}^{{\varvec{j}}}\right\}}_{i=1}^{N}$ be a set of MRI medical images under consideration for N number of patients. Each image ${{\varvec{y}}}_{i}={\left\{{y}_{i}^{J}\right\}}_{j=1}^{{n}_{i}}$ consists of a sequence of ${n}_{i}$ slices. The aim is to produce a valid classification for the stream of slices of images to produce a robust, interpretable classification model. The classification of individual slices often poses a complex challenge, particularly when these slices come from unknown statistical distributions. Common approaches to tackle this issue involve the use of deep learning algorithms combined with linear classifiers. While these methods can be effective, especially if a classifier is developed alongside the feature-dictionary, they tend to neglect critical details—specifically, the inherent similarities within classes and the relationships between different classes represented by coding coefficients. To address these shortcomings, a more specialized approach called Supervised within Class-similar Discriminative Dictionary Learning (SCDDL)^33^ has been formulated. SCDDL refines the classification process by constructing a specialized dictionary that captures the intrinsic similarities within each class through specific coding coefficients. These coefficients are meticulously engineered to echo the relationships between slices in the same category, enhancing the overall model's discriminatory power. Further precision is achieved by incorporating a linear classification error term, which guides the selection of the most optimal classifier for use with the established dictionary. Building on this foundation, the methodology has been extended into a more advanced form known as MKSCDDL^33^. This enhanced version integrates the concept of within-class similarity with kernel theory, facilitated by a multiple kernel fusion technique. This fusion allows for a more nuanced and granular differentiation between classes, capturing complex relationships that conventional methods may overlook.

Assuming that we have a training space A = [A_1_, A_2_, …, A_k_] $\in$ a 'd'-dimensional space consisting of 'k' classes, and let X be the coefficients obtained during the training of samples on the dictionary D. The model for SCDDL can be expressed as follows:

1 $$\begin{array}{c}\langle D,W,X\rangle =arg\underset{D,W,X}{min} \parallel A-DX{\parallel }_{F}^{2}+\alpha \parallel H-WX{\parallel }_{F}^{2}+\beta \parallel W{\parallel }_{F }^{2}\\ +{\lambda }_{1}\parallel X{\parallel }_{1}+{\lambda }_{2}\sum_{i=1}^{k} \left({\parallel{X}_{i}-{M}_{i}\parallel}_{F}^{2}+\eta {\parallel{X}_{i}\parallel}_{F}^{2}\right)\\ {\text{s}}{\text{u}}{\text{c}}{\text{h}} \, {\text{t}}{\text{h}}{\text{a}}{\text{t}} \, {\parallel{d}_{j}\parallel}_{2}^{2}=1, \, {\text{f}}{\text{o}}{\text{r}} \, {\text{a}}{\text{l}}{\text{l}} \, j=1,\dots ,m\end{array}$$

where $\parallel A-DX{\parallel }_{F}^{2}$: represents the Frobenius norm of the difference between $A$ and $DX$. $\parallel H-WX{\parallel }_{F}^{2}$: similar to the first term, this term aims to find $W$ and $X$ such that $WX$ approximates $H$. The $\alpha$ term is a weighting factor. $\parallel W{\parallel }_{F}^{2}$: this term is a regularisation term for $W$ using the Frobenius norm. $\beta$ is the regularisation parameter that controls the magnitude of $W$. $\parallel X{\parallel }_{1}$: this term is an L1 regularisation term for $X$, making the optimisation problem sparse. ${\lambda }_{1}$ is the regularisation parameter. $\sum_{i=1}^{k} \left({\parallel{X}_{i}-{M}_{i}\parallel}_{F}^{2}+\eta {\parallel{X}_{i}\parallel}_{F}^{2}\right)$ represents the within-Class-similar term. ${\lambda }_{2}$ and $\eta$ are the regularisation parameters. $\parallel {d}_{j}{\parallel }_{2}^{2}=1$ for all $j=1,\dots ,m$: this is a constraint on the columns ${d}_{j}$ of $D$, stating that they should be unit vectors in terms of the L2 norm.

With reference to the elastic net theory, the term ${\parallel{X}_{i}\parallel}_{F}^{2}$ combined with the term $\parallel X{\parallel }_{1}$ makes the Eq. (1) stable. Here, we consider $\eta =1$ for simplicity. Then the Eq. (1) can be reconstructed as follows:

2 $$\begin{array}{c}\langle D,W,X\rangle =arg\underset{D,W,X}{min} \parallel A-DX{\parallel }_{F}^{2}+\alpha \parallel H-WX{\parallel }_{F}^{2}\\ +\beta \parallel W{\parallel }_{F }^{2}+{\lambda }_{1}\parallel X{\parallel }_{1}+{\lambda }_{2}\sum_{i=1}^{k} \left({\parallel{X}_{i}-{M}_{i}\parallel}_{F}^{2}+{\parallel{X}_{i}\parallel}_{F}^{2}\right)\\ \\ \, {\text{s}}{\text{u}}{\text{c}}{\text{h}} \, {\text{t}}{\text{h}}{\text{a}}{\text{t}} \, \, {\parallel{d}_{j}\parallel}_{2}^{2}=1, \, {\text{f}}{\text{o}}{\text{r}} \, {\text{a}}{\text{l}}{\text{l}} \, j=1,\dots ,m\end{array}$$

Equation (2)'s optimisation approach has been examined in^34^ and demonstrated to increase the dictionary's discriminative categorisation.

The integration of Mercer kernels into the Sparse Classifier with Discriminative Dictionary Learning (SCDDL) algorithm, leading to the MKSCDDL extension, offers enhanced capabilities for handling high-dimensional data. Mercer kernels, such as linear and Gaussian kernels, facilitate the mapping of original feature space into a higher-dimensional space where linearly inseparable problems often become separable. Given $\phi (.)$ as a mapping function that transforms the original feature vectors into a higher-dimensional feature space, the kernelised version of the SCDDL algorithm can be defined by replacing the training sample vectors in Eq. (2) of the original SCDDL algorithm with their higher-dimensional counterparts $\phi (x)$. This extension allows the algorithm to exploit the geometric properties of the higher-dimensional space, potentially improving the classification performance.

The objective function for MKSCDDL is given by Eq. (3):

3 $$\begin{array}{c}\langle V,W,X\rangle =arg\underset{V,W,X}{min} \parallel \phi (A)-\phi (A)VX{\parallel }_{F}^{2}+\alpha \parallel H-WX{\parallel }_{F}^{2}\\ +\beta \parallel W{\parallel }_{F}^{2}+{\lambda }_{1}\parallel X{\parallel }_{1}\\ +{\lambda }_{2}\sum_{i=1}^{k} \left({\parallel{X}_{i}-{M}_{i}\parallel}_{F}^{2}\right. \left.+{\parallel{X}_{i}\parallel}_{F}^{2}\right)\\ \end{array}$$

By introducing the kernelised term $\parallel \phi \left(A\right)-\phi \left(A\right)VX{\parallel }_{F}^{2}$ MKSCDDL not only captures the non-linearities in the data but also combines multiple features into a unified dictionary learning framework.

### Scandent decision trees

The Scandent decision tree (SDT)^35^ addresses a crucial problem in multimodal classification tasks—namely, the nonuniformity of data. In many clinical or diagnostic settings, not all subjects have complete records with every possible feature or diagnostic marker. This could be due to a variety of reasons, including cost constraints or the advanced nature of certain diagnostic tests. The SDT model provides a robust solution to this issue by allowing for classification even when some features are missing from the records.

The SDT model works by first training a Support Decision Tree (DT) on a subset of the data where all features are available. This tree serves as the "gold standard" for classification based on all available attributes. In situations where all attributes are not available, the SDT comes into play. At each node in the SDT where a feature from the unavailable set is used for decision-making, a subtree ${T}_{i}$ is grown using only the available features (denoted by set $S$. The objective is to replicate, as closely as possible, the decision structure of the SDT using only the available attributes.

A major challenge in this approach is the potential sparsity of data at deeper levels of the SDT. As you go deeper into the decision tree, fewer records are available at each node, leading to less reliable and less accurate decisions. To counter this, a RF strategy is often employed. Multiple such subtrees are grown, and their outcomes are averaged or voted upon to form a more robust classification model. This ensemble approach helps mitigate the impact of decisions made based on sparse data. Furthermore, it has been shown that performance is enhanced^36^ when a feature enrichment strategy is employed. In this approach, the class labels produced by the subtrees ${T}_{i}$ are used to augment the feature set for records that only have features available from set $L$.This not only provides additional discriminative power but also improves the robustness of the classifier.

By addressing the nonuniformity in data availability, SDT offers a flexible and robust mechanism for classification tasks in settings where complete data may not always be available.

### Proposed methodology

**Figure 1:**
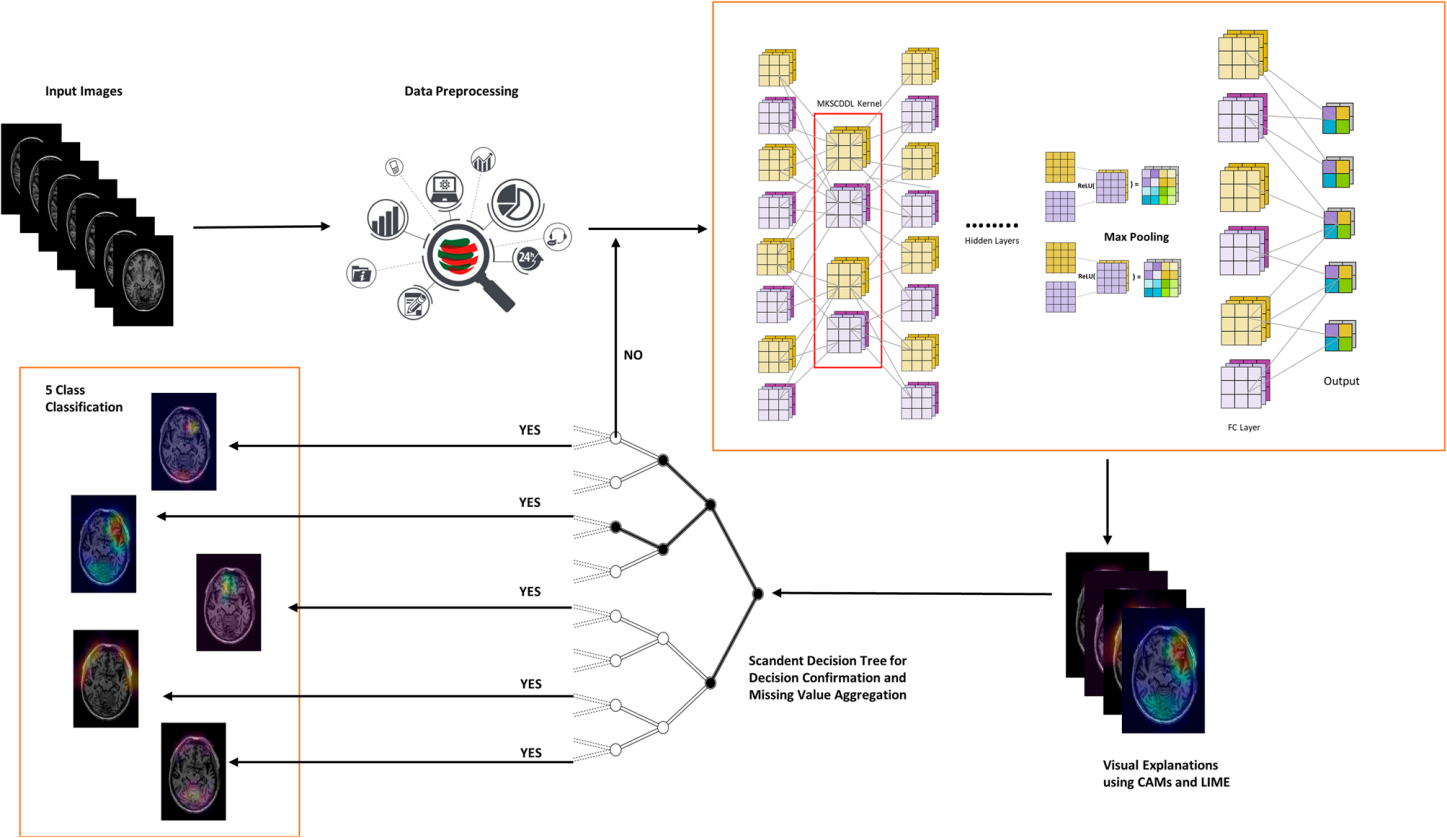
Proposed methodology.

We present an innovative framework aimed at achieving two critical objectives in medical image classification: high accuracy and interpretability. Our methodology uniquely integrates state-of-the-art techniques in CNNs, kernel methods, and explanation algorithms to classify medical images into five predetermined categories: AD, CN, MCI, Late-stage MCI, and early-stage MCI. The proposed framework is a composite of multiple components, each designed to address specific challenges in medical image classification and interpretation (see Fig. 1). Before feeding medical images into the neural network, we undertake a meticulous pre-processing routine. This involves dividing each image into "superpixels," which are clusters of pixels that share common characteristics. This segmentation enables more precise explanations later in the pipeline.

The core of our framework is a custom-designed CNN that has been trained to classify medical images into one of the five aforementioned classes. To further refine the classification process, we utilise the MKSCDDL kernel in the second layer of the CNN. This specialised kernel leverages the structural and anatomical similarities within images in the training set to minimise misclassification errors. By doing so, it produces a similarity measure that aids in categorising the images more accurately. A major challenge in using complex models like CNNs is the 'black box' nature, which makes it difficult to understand the rationale behind classifications. To counter this, we employ LIME and CAMs to construct a surrogate model. This interpretable model provides insights into why CNN classified a given image into a specific category.

Post-classification, the Scandent Decision Trees are used to segregate the tasks into appropriate categories. These decision trees validate the regions of interest within the images identified by LIME to ensure that the final classification aligns with the interpretive data. Lastly, we utilise a decision tree mechanism coupled with transfer learning techniques to confirm and enhance the classification accuracy. This dual-methodology approach ensures that the model is not only precise but also generalisable to new, unseen data. Through the integrated use of LIME and CAMs, we manage to generate an interpretable model that makes the decision-making process transparent and trustworthy, thereby instilling confidence in the medical practitioners who rely on this technology.

#### Dataset characteristics and pre-processing

The ADNI dataset is a comprehensive collection of MRI scans, each represented as a three-dimensional array consisting of 2D grayscale slices. Each slice has a fixed resolution of 256 × 256 pixels. However, the number of slices can differ from one patient to another, introducing an element of variability into the dataset. To make the dataset compatible with the experimental setup, all slices were standardised to a consistent resolution.

#### Partitioning of data for training and testing

The dataset was partitioned using an 80:20 ratio, where 80% of the data was used for training the model, and the remaining 20% was reserved for validation and testing. This partitioning strategy was carefully chosen to ensure that the model had sufficient data to learn the intricate features relevant to AD, MCI, and NC while also setting aside a robust subset for validation and performance evaluation.

#### Configuration of MKSCDDL kernel

Incorporated within the CNN, the MKSCDDL kernel was configured to operate as a linear kernel. To optimise its performance, a grid search technique was implemented. This allowed for fine-grained tuning of the kernel's weight parameters, with the search conducted in incremental steps of 0.1 to ensure precision.

#### Architecture and parameters of the CNN model

**Figure 2:**
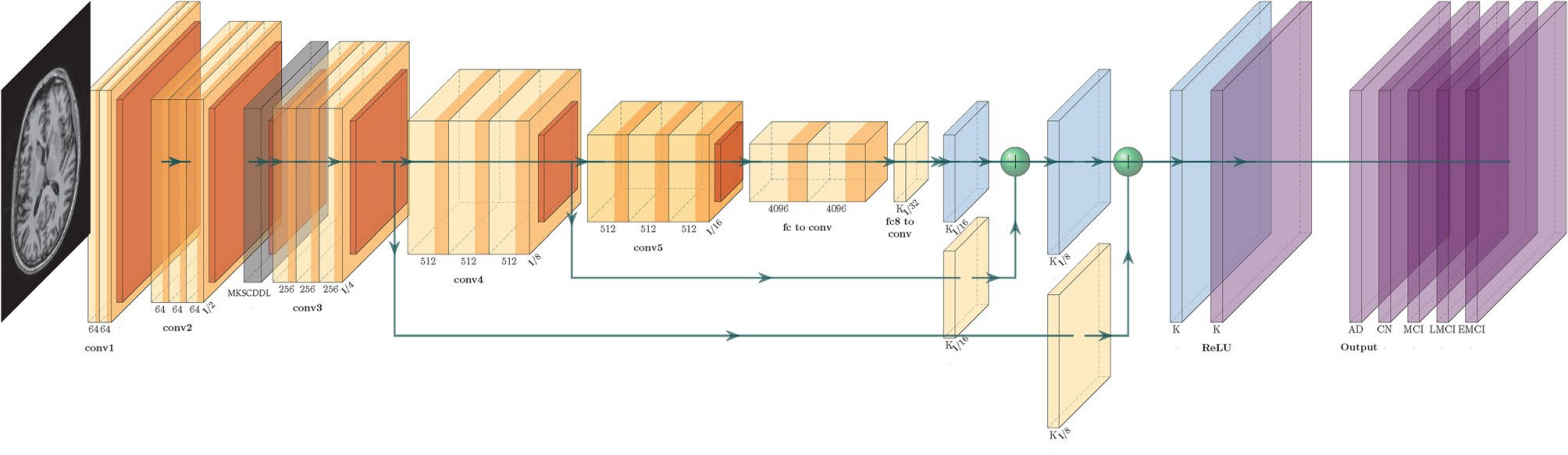
Architectural overview of the custom CNN (CNN + MKSCDDL).

The CNN model was architected to have five convolutional layers followed by two fully connected dense layers (see Fig. 2). Each convolutional layer was succeeded by a Rectified Linear Unit (ReLU) activation function to introduce non-linearity into the model. The CNN was trained from scratch using a learning rate of 0.001 and a batch size of 10, employing a consistent optimiser and loss function for uniformity in the training process. To further augment the feature extraction capabilities of the CNN model, a second Multi-Layer Perceptron (MLP) was introduced. This MLP was specially configured with the MKSCDDL kernel to work in tandem with the CNN, thereby creating a hybrid architecture that leverages the strengths of both neural networks and kernel methods.

#### Hyperparameters and optimisation strategy

To maintain a consistent and fair evaluation across different neural network architectures, we standardised the hyperparameters and initialisation procedures. Specifically, the Adam optimiser was chosen with a fixed learning rate of 10^–3^. A batch size of 10 was also consistently used across all experiments. In our proposed architecture, we deliberately restricted the use of data augmentation techniques to only include rotation. This cautious approach was taken to mitigate the risk of losing vital diagnostic information that could be crucial for accurate classification.

#### Pre-processing and multimodal feature fusion

Before deploying the ADNI dataset in our framework, a series of pre-processing steps were carried out. Our CNN model equipped with MKSCDDL kernels was used to merge multimodal feature sets. Features from MRI scans, along with class labels and clinical ratings, were initially extracted. At each layer within the kernel, different features were identified and subsequently aggregated for comprehensive analysis. The kernel moved or "strode" across the image data in distinct patterns at each layer, allowing each hidden layer to filter and spotlight specific features indicative of particular disorders.

#### Handling missing values with scandent trees

One of the critical challenges in medical imaging research is dealing with missing data, especially when multiple modalities like MRI, PET imaging, cognitive assessments, and various biomarkers are involved. To tackle this issue, we implemented the scandent tree methodology. This technique enhances classification accuracy by generating single-modality trees that mimic the feature space partitioning performed by a multimodal decision tree. The use of scandent trees enables the system to interpolate and fill in missing values, thereby improving the overall classification model's performance and robustness.

#### Training and evaluation procedures

All input images were resized to a uniform 256 × 256 resolution to ensure consistency. The processed MRI volumes generated volumetric distributions of AD distinct from MCI. We performed this differentiation by identifying occurrences of Gray Matter and White Matter in the brain scans and computing their respective probabilities. Saliency maps were then created based on these distributions to evaluate the model's classification consistency. These maps helped identify the brain regions most responsible for accurate disease classification. Figure 6 in our study delineates these critical regional areas, and Table 2 presents the quantitative metrics derived from these analyses.

**Table 2:** Comparison of classification results: CNN vs. CNN + MKSCDDL.

Target class	CNN				CNN + MKSCDDL			
	Accuracy (%)	Precision	Recall	F1-score	Accuracy	Precision	Recall	F1-score
AD	85.23	0.81	0.86	0.83	97.72	0.96	0.98	0.97
CN	69.54	0.68	0.63	0.66	84.45	0.89	0.87	0.66
EMCI	79.69	0.71	0.85	0.77	90.25	0.91	0.94	0.92
LMCI	92.35	0.94	0.98	0.96	95.36	0.96	0.98	0.97
MCI	86.69	0.91	0.70	0.79	93.75	0.97	0.93	0.94

#### Loss function and latent feature learning

In our proposed model, we advocate the use of a combined loss function—specifically, cluster loss and contrastive loss techniques—to encourage effective latent space learning. The model employs a categorical loss function, which stimulates the neural network to cluster latent features into semantically meaningful spaces. This innovative approach not only enhances the accuracy of the classification but also allows for the development of fine-grained models that can make highly specific predictions.

## Result analysis

### Comparative analysis

For our analysis, we used the Alzheimer's Disease Neuroimaging Initiative (ADNI) dataset, focusing on multiple evaluation metrics, including AUC, Receiver Operating Characteristic (ROC), accuracy, sensitivity, recall, and precision. To enhance the robustness and credibility of our results, we deployed a ten-fold cross-validation technique. In this approach, the dataset was partitioned into ten distinct subsets, each serving as a test set while the remaining subsets were used for training.

The comparative analysis between a Convolutional Neural Network (CNN) and the proposed model (see Table 2) offers illuminating insights into conditions including AD and various stages of cognitive impairment. The CNN + MKSCDDL model outpaces the CNN model across multiple key performance metrics—Accuracy, Precision, Recall, and F1-Score—indicating its superior capacity for predictive analysis. For instance, in diagnosing AD, the CNN + MKSCDDL model registers an impressive leap in accuracy, soaring from 85.23 to 97.72%, accompanied by an F1-Score jump from 0.83 to 0.97. This holds significant clinical relevance given the critical nature of early and accurate AD diagnosis. The enhanced model also proves its mettle in identifying Early and Late Mild Cognitive Impairment (EMCI and LMCI), showcasing marked improvements in Recall and F1-Score—metrics that are pivotal for capturing the maximum number of positive cases without inflating false positives. Specifically, for EMCI, the F1-Score ascends from 0.77 to 0.92, while for LMCI, it inches up from an already high 0.96 to 0.97.

Moreover, the advanced model significantly elevates the Recall in almost all categories, a critical improvement in medical settings where missing a positive case could lead to severe consequences. In the case of MCI, the Recall jumps remarkably from 0.70 to 0.93. This balanced performance across metrics, barring the anomaly in the CN category, underscores the CNN + MKSCDDL model's well-rounded capabilities. Thus, the fusion of CNN with MKSCDDL not only boosts the model's accuracy but also refines its balance between Precision and Recall, thereby advancing it a step closer to being a robust, clinically viable diagnostic tool for complex neurological conditions.

Table 3 presents a snapshot of the performance of various machine learning models in medical diagnostics, each rigorously evaluated on five key metrics: Accuracy, F1-Score, Sensitivity, Specificity, and AUC.

**Table 3:** Comparison with the existing models.

S. No	Model	Accuracy (%)	F1-Score	Sensitivity	Specificity	AUC
1	SCDDL-MRI	88.27	0.89	94.50	82.46	0.939
2	SCDDL-FDG- PET	91.18	0.93	86.40	95.61	0.970
3	SCDDL-florbetapir PET	85.64	0.84	85.50	85.61	0.937
4	MKL	93.64	0.95	96.20	91.23	0.963
5	MTFS	90.70	0.89	89.50	90.80	0.966
6	JRC	94.55	0.95	98.10	91.23	0.971
7	M2TFS	91.50	0.90	91.40	91.60	0.979
8	SVM	85.80	0.88	84.60	85.90	0.933
9	MDTC	88.40	0.87	87.20	88.50	0.950
10	Lasso	87.90	0.91	87.80	88.10	0.951
11	MDTL	94.70	0.95	94.10	94.80	0.988
12	U-Net	92.45	0.91	91.48	92.35	0.956
13	3DAN	86.12	0.89	87.33	89.32	0.912
14	VGGNet 3D	88.82	0.90	86.36	82.36	0.872
15	MaxNet	95.42	0.97	94.48	96.32	0.980
16	Explainable CNN + MKSCDDL (proposed)	98.27	0.97	98.87	96.46	0.982

We trained the models on 12,000 labelled MRI slices until convergence and subsequently tested them on a separate set of 4,600 MRI slices. Our comparative analysis, illustrated in Table 3, reveals that our proposed model substantially outperformed existing models like SCDDL-MRI, SCDDL-FDG-PET, and SCDDL-florbetapir PET in terms of accuracy and precision. The proposed Explainable CNN + MKSCDDL model emerges as the frontrunner, achieving an unparalleled accuracy of 98.27% and matching it with an AUC of 0.982. Its sensitivity and specificity figures, at 98.87% and 96.46%, respectively, indicate that the model excels in both identifying true cases and avoiding false alarms.

### Explainability analysis: unveiling deep model decision-making

Deep Learning (DL) methodologies have significantly impacted various scientific domains, notably healthcare, due to their ability to self-learn and generalise features. Despite their prowess, the "black-box" nature of these models has been a subject of concern, particularly when it comes to medical diagnoses. Our research makes strides in this direction by incorporating XAI techniques, specifically focusing on the interpretability of deep learning models when analysing brain scans.

#### Mapping salient brain regions for decision clarity

To mitigate the 'black-box' limitations of DL models, we manually mapped salient regions of the brain, as visualised in Fig. 5. By showcasing activated voxels in multiple 2D slices from diverse regions of the brain, we were able to statistically validate the significance of these regions in the decision-making process of the deep model. This makes a transparent layer to the AI algorithm, helping both clinicians and patients understand the rationale behind diagnostic decisions.

#### CN Individuals: stability in brain structures

**Figure 3:**
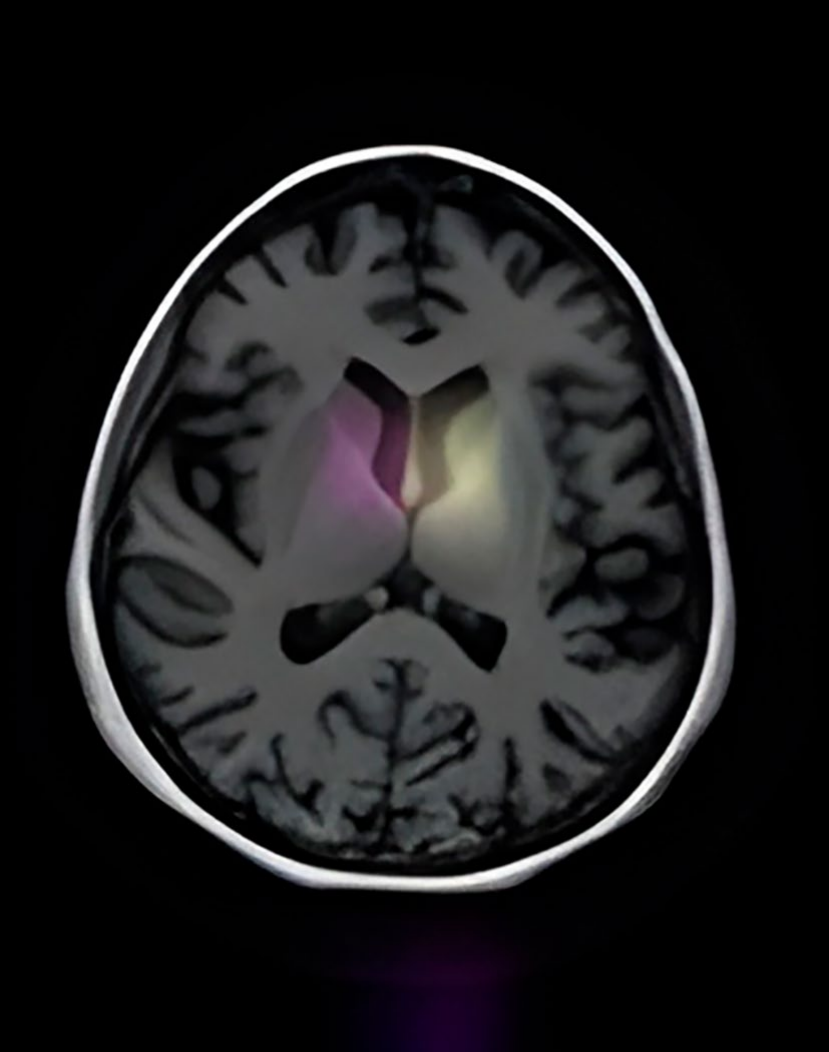
Scans of CN patients.

**Figure 4:**
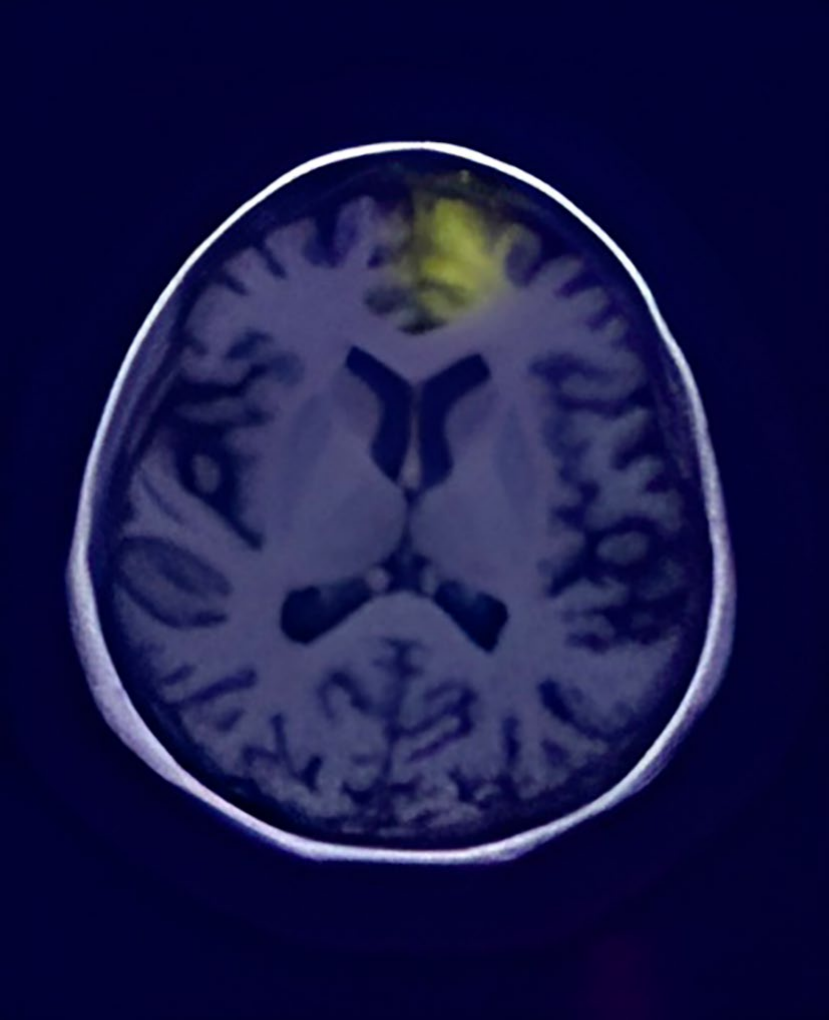
Scans of CN patients.

In Figs. 3, 4, and the first row of Fig. 6, we highlight the neural regions most pivotal in distinguishing CN individuals from AD patients. The key differentiating areas include the rostral Hippocampus, medial Amygdala, Globus Pallidus, lateral Amygdala, and the Parahippocampal gyrus. Remarkably, these regions remain stable over time in CN individuals, signifying a consistent state of cognitive health.

#### AD: dynamic changes in neural activities

**Figure 5:**
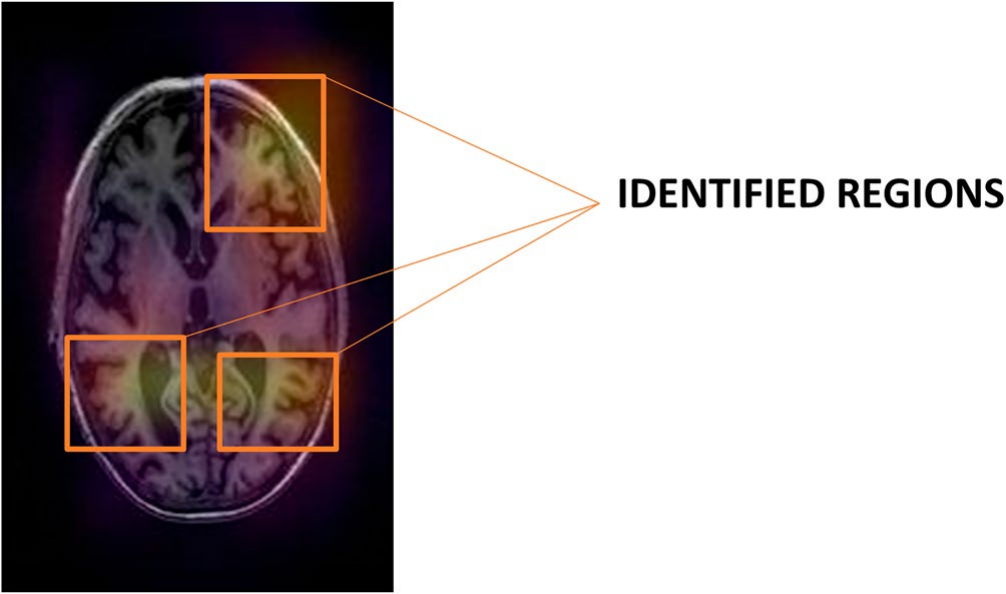
AD affected regions.

**Figure 6:**
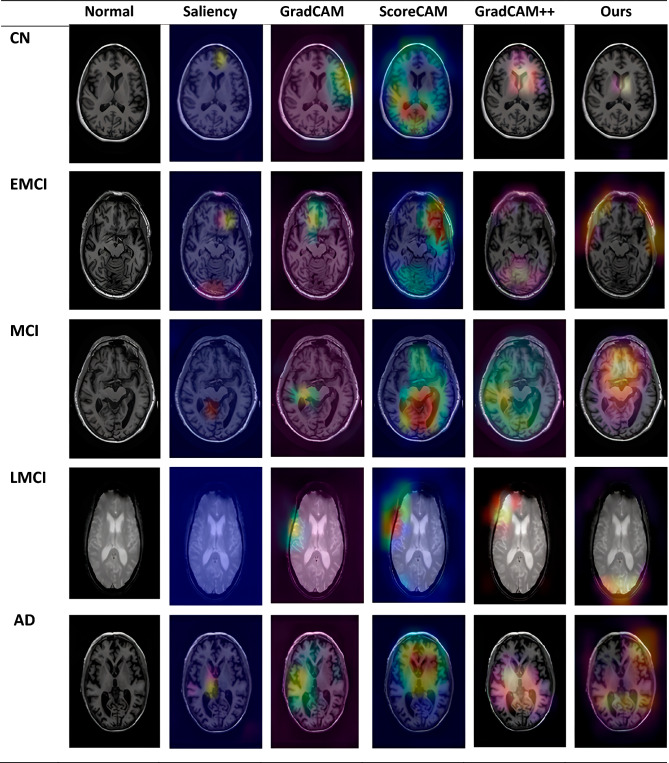
The ROI as highlighted with various CAMs to ensure a robust prediction using the heat map of model location identification concentration Row 1: CN, Row 2: EMCI, Row 3: MCI, Row 4: LMCI, Row 5: AD.

The bottom row of Figs. 5 and 6 focus on the AD scenario, illustrating how the disease manifests its impact across diverse brain regions. Notably, the regions most affected include the Hippocampus, medial and lateral Amygdala, posterior Hippocampus, dorsolateral putamen, rostroventral area, and Globus Pallidus. Our explainable model allows clinicians to observe these dynamically changing regions at each time point, thereby enabling more nuanced patient monitoring.

#### Tracking disease progression: from early MCI to late MCI

Our research goes a step further by scrutinising disease progression from EMCI to LMCI. Panels 2, 3, and 4 of Fig. 6 reveal that patients progressing from EMCI to LMCI undergo more rapid neurodegeneration than those already diagnosed with advanced AD. The network analysis used in our study identifies similar impacted regions in both MCI and converted EMCI patients, including the middle and lateral Amygdala, Parahippocampal region, and Hippocampus. However, as the disease progresses in converted EMCI patients, additional regions show signs of impairment, such as the caudal Hippocampus and dorsolateral putamen.

### Ablation studies

Ablation studies are crucial for understanding the performance contributions of different components in a machine learning model. In the context of our CNN model for image classification, we conducted a comprehensive ablation study to evaluate the impact of various hyperparameters and architectural choices on model performance. The base model was established with Adam optimiser and MKSCDDL kernels. The performance metrics under consideration were Accuracy, F1-Score, and Recall. Various experiments were conducted by modifying one or a combination of elements, and the metrics were recorded in Table 4.

**Table 4:** Ablation studies.

S. no.	Experiment description	Accuracy (%)	F1-score	Recall	Sensitivity
1	Base model (Adam optimizer, MKSCDDL kernels)	98.27	0.97	0.988	98.87
2	Change to RMSprop optimizer	97.85	0.97	0.978	96.40
3	Change to SGD optimizer	95.20	0.94	0.952	95.50
4	Remove one Conv2D layer (originally with 32 filters)	97.10	0.96	0.97	96.20
6	Combination of SGD and extra Conv2D layer	97.40	0.97	0.974	97.50
6	Add a new dropout layer (0.3)	98.10	0.97	0.981	98.10
7	Replace ReLU with LeakyReLU in one layer	98.25	0.97	0.974	97.72
8	Add L2 regularization to dense layers	97.80	0.97	0.977	87.20
9	Remove MKSCDDL kernels	95.00	0.93	0.95	87.80
10	Use MaxPooling instead of GlobalAveragePooling	97.50	0.96	0.975	94.10
11	Increase batch size to 20	98.10	0.97	0.98	91.48
12	Decrease batch size to 5	97.70	0.97	0.976	87.33
13	Use Swish activation in one layer	98.40	0.97	0.983	86.36
14	Use ELU (exponential linear unit) activation in one layer	98.20	0.97	0.982	94.48
15	Increase learning rate to 0.001	97.90	0.97	0.979	97.80
16	Decrease learning rate to 0.00001	98.00	0.97	0.980	98.12
17	Remove all dropout layers	97.20	0.96	0.972	97.15
18	Add a Gaussian noise layer	98.25	0.97	0.982	98.20

Table 4 presents an exhaustive ablation analysis meticulously designed to explore the repercussions of various architectural and hyperparameter modifications on the performance of the machine learning model. Focused on four pivotal metrics—Accuracy, F1-Score, Recall, and Sensitivity—this empirical investigation furnishes nuanced insights into the model's robustness and susceptibility to changes.

Commencing with the base model, which employs Adam Optimizer in conjunction with MKSCDDL kernels, the performance is unequivocally superior, boasting an accuracy of 98.27%, an F1-Score of 0.97, and near-flawless recall and sensitivity rates of 0.988 and 98.87% respectively. This serves as a high-performance baseline against which all other configurations are compared.

The ablation study investigates the ramifications of substituting the Adam Optimizer with RMSprop and Stochastic Gradient Descent (SGD). Here, the Adam Optimizer emerges as the optimally efficient choice, with RMSprop and SGD trailing with accuracies of 97.85% and 95.20%, respectively. The marginal decline in these metrics accentuates the efficacy of the Adam Optimizer in this specific modelling context.

Structural alterations to the model's architecture, such as the removal of a Conv2D layer initially possessing 32 filters, resulted in a moderate decrement in performance, with the accuracy dropping to 97.10%. This indicates the layer's non-trivial contribution to the model's superior discriminative capability.

Moreover, when the activation functions were substituted—from ReLU to LeakyReLU—the model's accuracy decreased marginally, reflecting the subtle yet impactful role of activation functions in neural networks. On the other hand, the removal of MKSCDDL kernels led to a more drastic reduction in accuracy, plummeting to 95.00%. This substantiates the kernels' pivotal role in enhancing the model's capacity for nuanced classification.

In addition to architectural changes, the study delved into the impact of varying batch sizes and learning rates. While these changes did not result in drastic fluctuations in performance metrics, they did offer incremental improvements or reductions, thereby emphasising the need for fine-tuned hyperparameter selection.

The ablation study fortifies our confidence in the base model, characterised by the Adam Optimizer and MKSCDDL kernels, as the most judicious selection for this application. Its exceptional performance across a multitude of metrics not only attests to its robustness but also underscores its comprehensive applicability. The remarkable discriminative power endowed by the MKSCDDL kernels emerges as a cornerstone for the model's high performance, thereby validating our decision to adopt this particular configuration.

### Inferences: bridging the gap between data and diagnosis

#### Multimodal data fusion for robust classification

Our experimental setup leveraged a rich dataset comprised primarily of MRI scans, with additional PET scans, to distinguish among AD, MCI, and CN individuals. A key strength of our methodology is the integration of multiple data modalities via the scandent decision tree algorithm. This innovative approach enables the filling of missing data points, thereby enhancing the robustness and trustworthiness of the classifier. Unlike prior research that often sidestepped the issue of missing values, our study employed explainable techniques to identify critical brain regions, thereby lending verifiable credence to our predictions.

#### Identifying vulnerable brain regions in AD

Concurring with extant literature, our research pinpoints specific brain regions—such as the Basal Ganglia, Amygdala, Parahippocampal Gyrus, and Hippocampus—as being disproportionately affected by AD. These regions are instrumental in various cognitive and emotional functions. Thus, their impairment manifests in the multifaceted symptoms observed in AD patients.

#### Neural activation patterns: a discriminatory marker

Our proposed neural network succeeds in differentiating between CN individuals and those with AD through unique activation patterns, especially in the dorsolateral Putamen regions. Such patterns were exclusively observed in AD patients and remained absent in CN individuals. Within the AD cohort, consistent activation of the Amygdala and Hippocampus was noted, indicative of their central role in AD pathology.

#### Emotional and cognitive dysregulation in AD

Interestingly, our results show that alterations in the Amygdala are closely associated with heightened feelings of anger and anxiety among AD patients. Furthermore, we identified other subcortical areas, such as the Parahippocampal Gyrus, Thalamus, and Putamen, as being significantly activated in AD. These regions govern a wide range of functions, including cognition, motor skills, and sensory perception—all of which are compromised in AD.

#### The Precuneus and decision-making deficits

Our research underscores the deterioration of the Precuneus region, located within the Parietal lobe, as a defining feature of AD. In addition, we found that the Thalamus and Putamen regions showed marked degradation in AD patients. This decline is of significant concern as it affects critical faculties like problem-solving and decision-making, thereby contributing to behavioural issues such as apathy and obsessive tendencies.

#### Implications for treatment and intervention

By elaborating on the complex neural underpinnings of AD, this study underscores the urgency for targeted interventions. Understanding the specific brain regions and functionalities affected by AD could pave the way for more precise and effective treatments.

## Conclusions and future directions

Timely identification and intervention in cognitive disorders like AD and MCI are pivotal for the effective clinical management of these conditions. The study presented herein contributes to this critical need by introducing a cutting-edge system that melds the power of advanced deep learning algorithms with the transparency of XAI. Our methodology utilises a CNN with an MKSCDDL algorithm. By using the structural and anatomical patterns that can be seen in linked neuroimaging data, this fusion greatly improves the classification accuracy of the model.

Crucially, the integration of LIME and CAM endows our model with a level of transparency and interpretability that is often elusive in deep learning architectures. This explainability is invaluable for healthcare practitioners, as it allows them to identify specific brain regions that are most likely affected by cognitive disorders. Our model was rigorously evaluated using the Alzheimer's Disease Neuroimaging Initiative (ADNI) dataset and was pitted against a standard CNN model for benchmarking. The actual results are readily apparent: our method is better than the traditional one in key measures like precision, accuracy, and recall. The saliency maps, generated via LIME and CAM, further enrich our understanding of AD's underlying mechanisms.

While our results are promising, the journey towards optimal cognitive disease diagnosis is far from over. Future iterations of our work will investigate the potential benefits of incorporating alternative kernel functions to amalgamate multimodal data more effectively. We are also keen to explore the incorporation of different imaging modalities to broaden the scope of our diagnosis. Additionally, the utilisation of other advanced deep learning architectures, coupled with transfer learning techniques, stands to further elevate the model's performance metrics. In conclusion, our research signifies a pivotal step forward in the clinical diagnosis and management of cognitive diseases. By harmonising high computational power with transparent decision-making, we offer the medical community a reliable, accurate, and intuitive tool that has the potential to revolutionise cognitive healthcare.

## Data Availability

The data is publicly available to use on the ADNI website.

## References

[1] Gao Y (2016). ZCWPW1 is associated with late-onset Alzheimer’s disease in Han Chinese: A replication study and meta-analyses. Oncotarget.

[2] Selkoe DJ, Hardy J (2016). The amyloid hypothesis of Alzheimer’s disease at 25 years. EMBO Mol. Med..

[3] Scheltens P (2021). Alzheimer’s disease. Lancet.

[4] Knopman DS (2021). Alzheimer disease. Nat. Rev. Dis. Prim..

[5] Petersen RC (2001). Practice parameter: Early detection of dementia: Mild cognitive impairment (an evidence-based review). Neurology.

[6] Garcés P (2014). The default mode network is functionally and structurally disrupted in amnestic mild cognitive impairment—A bimodal MEG-DTI study. Neuroimage (Amst)..

[7] Petersen RC (2004). Mild cognitive impairment as a diagnostic entity. J. Intern. Med..

[8] Tan MS (2013). NLRP3 polymorphisms are associated with late-onset Alzheimer’s disease in Han Chinese. J. Neuroimmunol..

[9] Álvarez-Miranda E, Farhan H, Luipersbeck M, Sinnl M (2017). A bi-objective network design approach for discovering functional modules linking Golgi apparatus fragmentation and neuronal death. Ann. Oper. Res..

[10] Suk II H, Lee SW, Shen D (2015). Latent feature representation with stacked auto-encoder for AD/MCI diagnosis. Brain Struct. Funct..

[11] Wang P (2016). Multimodal classification of mild cognitive impairment based on partial least squares. J. Alzheimers. Dis..

[12] Tanveer M (2020). Machine learning techniques for the diagnosis of Alzheimer’s disease. ACM Trans. Multimed. Comput. Commun. Appl..

[13] Ortiz A, Munilla J, Górriz JM, Ramírez J (2016). Ensembles of deep learning architectures for the early diagnosis of the Alzheimer’s disease. Int. J. Neural Syst..

[14] Nanni L, Brahnam S, Salvatore C, Castiglioni I (2019). Texture descriptors and voxels for the early diagnosis of Alzheimer’s disease. Artif. Intell. Med..

[15] Feng J, Zhang SW, Chen L (2020). Identification of Alzheimer’s disease based on wavelet transformation energy feature of the structural MRI image and NN classifier. Artif. Intell. Med..

[16] Leming M, Das S, Im H (2022). Construction of a confounder-free clinical MRI dataset in the Mass General Brigham system for classification of Alzheimer’s disease. Artif. Intell. Med..

[17] Su J, Shen H, Peng L, Hu D (2021). Few-shot domain-adaptive anomaly detection for cross-site brain images. IEEE Trans. Pattern Anal. Mach. Intell..

[18] Pan Y, Liu M, Xia Y, Shen D (2022). Disease-image-specific learning for diagnosis-oriented neuroimage synthesis with incomplete multi-modality data. IEEE Trans. Pattern Anal. Mach. Intell..

[19] Basaia S (2019). Automated classification of Alzheimer’s disease and mild cognitive impairment using a single MRI and deep neural networks. NeuroImage Clin..

[20] Lei B (2020). Deep and joint learning of longitudinal data for Alzheimer’s disease prediction. Pattern Recognit..

[21] Lian C, Liu M, Zhang J, Shen D (2020). Hierarchical fully convolutional network for joint atrophy localisation and Alzheimer’s disease diagnosis using structural MRI. IEEE Trans. Pattern Anal. Mach. Intell..

[22] Jain R, Jain N, Aggarwal A, Hemanth DJ (2019). Convolutional neural network based Alzheimer’s disease classification from magnetic resonance brain images. Cogn. Syst. Res..

[23] Spasov S, Passamonti L, Duggento A, Liò P, Toschi N (2019). A parameter-efficient deep learning approach to predict conversion from mild cognitive impairment to Alzheimer’s disease. Neuroimage.

[24] Wang H (2019). Ensemble of 3D densely connected convolutional network for diagnosis of mild cognitive impairment and Alzheimer’s disease. Neurocomputing.

[25] Cheng B (2015). Multimodal manifold-regularised transfer learning for MCI conversion prediction. Brain Imaging Behav..

[26] Suk II H, Wee CY, Lee SW, Shen D (2016). State-space model with deep learning for functional dynamics estimation in resting-state fMRI. Neuroimage.

[27] Li F, Liu M (2018). Alzheimer’s disease diagnosis based on multiple cluster dense convolutional networks. Comput. Med. Imaging Graph..

[28] Essemlali A, St-Onge E, Descoteaux M, Jodoin P-M (2020). Understanding Alzheimer disease’s structural connectivity through explainable AI. Proc. Mach. Learn. Res..

[29] El-Sappagh S, Alonso JM, Islam SMRR, Sultan AM, Kwak KS (2021). A multilayer multimodal detection and prediction model based on explainable artificial intelligence for Alzheimer’s disease. Sci. Rep..

[30] Yu L, Xiang W, Fang J, Phoebe Chen YP, Zhu R (2022). A novel explainable neural network for Alzheimer’s disease diagnosis. Pattern Recognit..

[31] Lombardi A (2022). A robust framework to investigate the reliability and stability of explainable artificial intelligence markers of mild cognitive impairment and Alzheimer’s disease. Brain Inform..

[32] Shojaei S, Saniee Abadeh M, Momeni Z (2023). An evolutionary explainable deep learning approach for Alzheimer’s MRI classification. Expert Syst. Appl..

[33] Wu X, Li Q, Xu L, Chen K, Yao L (2017). Multi-feature kernel discriminant dictionary learning for face recognition. Pattern Recognit..

[34] Xu L (2016). Prediction of progressive mild cognitive impairment by multi-modal neuroimaging. Biomarkers.

[35] Hor S, Moradi M (2015). Scandent tree: A random forest learning method for incomplete multimodal datasets. Lect. Notes Comput. Sci. (including Subser. Lect. Notes Artif. Intell. Lect. Notes Bioinform.).

[36] Hor S, Moradi M (2016). Learning in data-limited multimodal scenarios: Scandent decision forests and tree-based features. Med. Image Anal..

